# The Evolving Landscape of Gout in the Female: A Narrative Review

**DOI:** 10.3390/gucdd2010001

**Published:** 2023-12-30

**Authors:** Jennifer Lee, Nicholas Sumpter, Tony R. Merriman, Ru Liu-Bryan, Robert Terkeltaub

**Affiliations:** 1 Department of Internal Medicine, Division of Rheumatology, Seoul St Mary’s Hospital, College of Medicine, The Catholic University of Korea, Seoul 06591, Republic of Korea; 2 Division of Clinical Immunology and Rheumatology, University of Alabama at Birmingham, Birmingham, AL 35294, USA; 3 Department of Microbiology and Immunology, University of Otago, Dunedin 9054, New Zealand; 4 VA San Diego Healthcare System, San Diego, CA 92161, USA; 5 Division of Rheumatology, Autoimmunity and Inflammation, Department of Medicine, University of California San Diego School of Medicine, La Jolla, CA 92093, USA

**Keywords:** gout, hyperuricemia, ABCG2, SLC2A9, urate transporter, alcohol, fructose, COVID-19, chronic kidney disease, hypertension

## Abstract

Gout is at least three times more prevalent in males than in females. However, concurrent with rising total gout prevalence, complex factors, including comorbidities, diet, lifestyle, and aging, have promoted higher gout prevalence in females. This narrative review focuses on summarizing recent developments in the landscape of gout in females and the mechanisms involved. New knowledge on sex hormone effects on both urate-excreting and urate-reabsorbing transporters and higher hypertension and chronic kidney disease prevalence in females compared to males may help explain why gout incidence rises robustly after menopause in females, to approach that in males. Racial and ethnic factors, risk profiles based on heritable genetic polymorphisms of urate transporters, diet, body mass index, and lifestyle factors differ according to sex. In addition, sex differences in clinical phenotypes, outcomes of gout, and non-gout illnesses include more frequent comorbidities, more pain and disability during gout flares, different perceptions of disease burden, and more frequent severe cutaneous hypersensitivity reaction to allopurinol in females. Collectively, such findings support the potential clinical benefits of tailoring gout and hyperuricemia treatment according to sex.

## 1. Introduction

Gout is a highly prevalent inflammatory joint disease that develops in individuals with hyperuricemia, with consequent tissue deposition of monosodium urate (MSU) crystals [1,2]. In the USA, the most recent published gout prevalence is estimated at 5.1% of adults, with ~12 million adults affected by the disease [3]. Urate, the end product of purine metabolism, is normally excreted mainly (~70%) by the kidney and to a lesser degree (~30%) via transport into the gut. Multiple heritable and acquired factors that regulate urate transport in the kidney and the gut and affect urate production modulate urate home-ostasis and promote hyperuricemia [4–8]. MSU crystal deposition is promoted by factors including altered articular tissue extracellular matrix homeostasis [9,10]. The crystals can trigger an inflammatory cascade mediated in large part by NLRP3 inflammasome activation and the release of IL-1 [1,11], thereby resulting in acute flares of inflammatory arthritis superimposed on chronic synovitis [12,13]. A mixed granulomatous, fibrotic, chronic tissue inflammatory reaction to MSU crystal deposits mediates the formation of articular and subcutaneous tophi and the progression to erosive joint disease [12,14].

Gout remains a male-predominant disease, with male-female sex ratio at least 3:1 [2,15–21]. However, the sex difference for prevalence narrows in association with a sharp rise of incident gout in females after menopause [22]. For example, the male to female ratio was only 2.3 in those over 70 years (yrs)of age in a study conducted in the U.K. [23]. Since gout in females may be less well recognized clinically [24], gout is possibly under-reported in females. Regardless, at least ~5% of elderly females in the USA self-reported the diagnosis of gout [25]. Of all female gout patients, only 1–4.5% have premenopausal onset [23,26]. In these uncommon cases, renal dysfunction such as nephropathic effects induced by calcineurin inhibitors and interstitial nephropathies due to analgesic abuse, lead nephropathy, and strong genetic risk factor(s) are typically the driving force in developing hyperuricemia and gout [27].

Gout has increased in incidence over the last few decades in the USA, many other developed countries, and many less well-developed nations [2,3,17,28]. Multiple studies [4,15,23,29–56] have detailed and illuminated the epidemiology and risk factors for gout in females. Different clinical characteristics in female gout have been reported [6,46,49]. However, the precise mechanisms that drive these sexual differences still remain to be elucidated. This narrative review summarizes these and other recent developments, provides pathophysiologic perspectives, and addresses the remaining questions in the landscape of gout in females. The literature cited was chosen from MEDLINE English language literature searches. The search strategy aimed to identify, particularly but not exclusively for the last 7 years, relevant papers published on gout, urate handling, and sex differences, using combination terms of ‘gout’, ‘sex’, ‘women’, ’uric acid’, ’female’.

## 2. Mechanisms That Decrease the Gout and Hyperuricemia Sex Ratio after Menopause

### 2.1. Serum Urate Levels According to Sex

Overall, serum urate is unequivocally lower in females than males [57]. For example, in the USA, mean serum urate in males was recently estimated at 6.04 mg/dL, compared to 4.79 mg/dL in females [58]. In a recent Austrian study, females with an average age of 51 had a mean serum urate 4.10 ± 1.15 mg/dL vs. 5.29 ± 1.2 mg/dL in males with an average age of 41 [59]. Female gout patients develop gout at an older age than male patients, and female gout patients tend to have more comorbidities that promote hyperuricemia, most notably so for hypertension (HTN) and chronic kidney disease (CKD) [55]. Concordantly, the sex difference of serum urate lessens with increasing age, but especially so after menopause [60]. For example, in a study of 58,870 Korean females, the prevalence of hyperuricemia (defined as >6.0 mg/dL in females) was 2.7% in the pre-menopause population and 6.7% in the post-menopause population [61].

A substantial limitation of many studies of serum urate in females with hyperuricemia is estimated based on standard deviations from the mean for serum urate in females, with “hyperuricemia” defined at a lower serum urate in females than males. In this regard, National Health and Nutrition Examination Survey (NHANES) data from 2015–2016 estimated the prevalence of gout in males to be 5.2% [4.4–6.2%] and 2.7% [2.0–3.8%] in females [58]. In that study, when hyperuricemia was defined as >7.0 mg/dL, only 4.2% [3.3–5.3%] of females were hyperuricemic compared to 20.2% [16.6–24.3%] of males. In our opinion, the conventional physiologic definition of hyperuricemia (greater than either 6–8 or 7.0 mg/dL), based on the predominant evidence for limited urate solubility in a physiologic solution, should be applied universally to both sexes to support biologic rigor in studies in this field and to allow direct translatability to gout.

### 2.2. Effects of Sex Hormones on Urate Transporters

The forces driving male predominance in gout prevalence start with differences in androgen and estrogen sex hormone effects [62]. In this light, medical androgen deprivation therapy lowered serum urate in prostate cancer patients [63]. Moreover, testosterone administration in female to male transgender individuals significantly increases serum urate compared to baseline at 2 years [64]. Progesterone level was negatively correlated with serum urate level in premenopausal females, whereas follicle stimulating hormone (FSH) was positively correlated with serum urate level [65]. Differences in sex hormones lessen after menopause, and postmenopausal hormone replacement therapy in females decreases the risk of incident gout [44].

The uricosuric effects of estradiol appear substantial. In this context, estrogen administration to male to female transgender people not undergoing orchiectomy is associated with decreased serum urate and increased renal urate fractional excretion [64]. Mechanistically, estrogen suppresses the murine kidney protein levels of urate-reabsorbing transporters such as urate transporters URAT1 and GLUT9 [66]. Estrogen downregulates GLUT9, at least partly, post-transcriptionally via estrogen receptor (ER)-beta induced proteasomal degradation [67].

Serum urate-regulating effects of sex hormones are not limited to renal tubule transporters (Table 1). A prime example is that estradiol upregulates intestinal ATP binding cassette subfamily G member 2 (ABCG2) expression through the phosphoinositide 3-kinase (PI3K)/Akt pathway [68]. ABCG2 is a renal and gut epithelial cell-expressed urate-excreting transporter. ABCG2 exerts major regulatory effects by the common ABCG2 variant Q141K encoded by ABCG2rs2231142 [69] on the heritable risks of hyperuricemia, gout, early-onset gout, and tophaceous disease [70–81]. Much of the regulation of serum urate by ABCG2 occurs by effects on urate transport into the gut. Other extra-renal effects of urate transporters (e.g., GLUT9, ABCC4) could also regulate urate metabolism and circulating urate levels in the intestine and/or liver [82,83].

Heritability of serum urate level is estimated to be 30–60%, with major effects of single nucleotide polymorphism (SNP)s in multiple urate transporter genes [7,84–86]. However, such contributions, reflected in the effect size of certain genes, differ according to sex. A prime example is SNPs in SLC2A9 (e.g., rs7442295, rs734553), which have greater effect sizes in females, whereas ABCG2 SNPs rs2231142 and rs2199936 have greater effect sizes in males [87,88].

### 2.3. Sex Hormones in Purine Metabolism

Purine metabolism in females with gout is similar to that in males with primary gout, including decreased renal clearance and fractional excretion of urate, hypoxanthine, and xanthine and increased mean plasma urate, hypoxanthine, and xanthine levels [93]. However, plasma xanthine oxidoreductase (XOR) activity, measured in patients with coronary artery spasm, was reported to be significantly lower in females [94]. The difference could be due to estradiol and other sex hormones. In this context, estradiol stereoisomers prevented a hypoxia-induced increase in XOR enzymatic activity at a posttranscriptional level by a receptor-independent mechanism in cultured microvascular endothelial cells [95]. In addition, the genes of two major enzymes in purine metabolism—hypoxanthine-guanine phosphoribosyltransferase (HPRT) and phosphoribosylpyrophosphate synthetase—are on the X chromosome, and associated with X-linked inborn errors of purine metabolism (also phosphoribosylpyrophosphate synthetase superactivity and HPRT deficiency including Lesch-Nyhan disease) [96,97]. Generally, female heterozygote carriers do not develop symptoms unless their normal alleles are inactivated due to skewed X chromosome inactivation, while males with pathogenic variants generally are affected.

XOR and urate transporter activity in the prostate is an obvious distinction between the sexes. A positive correlation was discovered between XOR activity and prostate-specific antigen levels in the serum in prostate cancer patients [98]. Human single cell RNA sequencing data showed that XOR is expressed in prostatic basal and urothelial cells, and urate concentration is robust in the murine prostate [99]. However, patients who underwent prostatectomy alone for cancer showed a non-significant change in serum urate, though there was a decrease in those patients with hyperuricemia [63]. Notably, following castration for prostate cancer, serum urate falls in a transitory way [100], and androgen deprivation therapy lowers serum urate in prostate cancer patients [63]. Hence, the role of the prostate by itself in sex differences for serum urate requires further investigation.

## 3. Sex Differences in Risk Factors for Gout

### 3.1. Age, Race, Ethnicity Demographic Factors

Age is a well-established risk factor for gout [101–103]; female gout patients are older than males on average, and the hazard ratio for incident gout with every one-year increase is higher in females than males [104,105]. Black females have a higher risk for developing gout than white females [53], suggesting the role of ethnicity as a risk factor. In accordance with this, a nation-wide study showed that emergency department visits (relative ratio 5.91 [5.79–6.03] and hospitalization (relative ratio 4.80 [4.45–5.17] were strikingly higher in Black than White females [106]. However, a recent study using NHANES 2007–2016 showed that the effect of ethnicity diminished or became attenuated after adjusting for potential confounders including low income and low education [15]. Therefore, associated diet, social determinants, and clinical factors rather than ethnicity per se appear to contribute to higher incident gout risk in Black females.

Notably, prevalence of hypertension in the USA in 2017–2020 was 57.5% in Black males and was 58.4% in Black females compared to 48.9% in White males and 42.6% in White females [107]. Moreover, the prevalence of gout in those of East and South Asian descent residing in the USA has steeply increased and numerically exceeded all other ethnicities in the 2017–2018 period [3]. However, gout prevalence in females was comparable among ethnicities, whereas gout prevalence of Asian males was the highest of all studied ethnicities and races after adjustment for social and clinical factors [3].

### 3.2. Diet, Obesity, Alcohol, Smoking

Risk factors for gout include obesity, alcohol consumption, high fructose consumption [101], and foods such as meat and seafood [108]. Though alcohol consumption in female gout patients is higher than in controls, the effects of alcohol on the risk of gout in females are lower than in males [52]. In this context, a recent Japanese study found that alcohol consumption is a risk factor for hyperuricemia or gout in males but not in females. Furthermore, in the same study, smoking increased the risk for gout only in females [45].

Female gout patients have a higher frequency of obesity compared to male patients. In one study, the risk of gout was reported to be higher in females with body mass indexes (BMI) ≥27 kg/m^2^ (adjusted relative risk [RR] 1.30 in males and 2.15 in females) [104]. In another study using linear Mendelian randomization (MR), one standard deviation higher BMI increased the incidence rate for gout (incidence rate ratio [IRR] = 1.73, 95% confidence interval [CI] [1.56–1.92]) in males and females. That said, BMI was found to be a stronger risk factor for gout in females compared to males (*p* = 0.0043). Nonlinear effects of BMI were identified for gout in both males and females, but nonlinearity for gout was more pronounced in males compared to females (*p* = 0.03) [109]. This reflects a stronger causal effect of BMI on gout in leaner people.

Associations with gout of food, lifestyle factors, and genetic predisposition in genome-wide association studies (GWAS) [7,47,79,85,86,110–118] have prompted the study of relative contributions to incident gout of non-modifiable genetic and modifiable risk factors [119]. Two previous studies, which included male subjects (50%, ~75%, respectively), showed a relatively small contribution of dietary factors to serum urate levels [120,121]. In a prospective cohort study limited to female subjects, subjects with less healthy diets (low Dietary Approaches to Stop Hypertension [DASH] score) had higher risks of incident gout than those with healthy diets, but this was much more prominent in those with increased genetic predispositions (high genetic risk score [GRS]) [42]. Significantly, 51% of the excess risk of incident gout was attributable to the additive gene–diet interaction in the cohorts studied [73].

### 3.3. Comorbidities

Table 2 summarizes gout comorbidities as risk factors for gout development, comparing sex differences where available. Gout comorbidities that promote hyperuricemia include hypertension, metabolic syndrome/insulin resistance, obesity, type 2 diabetes mellitus (T2DM), CKD, and heart failure (HF) [122]. Observational studies suggest that the incidence of gout is increased in hypertensive patients in both males and females [6,55]. However, MR studies have had conflicting results. An analysis of the Taiwan biobank found that the liability of hypertension does not have a causal effect on gout [123]. On the other hand, a genetic analysis of over one million European ancestors found that systolic blood pressure and pulse pressure had a causal effect on serum urate and gout, but sex-specific effects were not identified [124]. Observational studies showed that HF or hypertension is more common in female gout patients [21]. This may be a result of the higher frequency of urate-elevating diuretic use in female gout patients. In this regard, an MR study found no consistent evidence for the causal effect of HF on serum urate levels [125].

T2DM is more prevalent in female gout patients [52], and female gout patients are at higher risk than males for developing T2DM [39]. However, T2DM itself was not a causal factor of incident gout in two MR studies [124,126], though there is evidence by MR for a causal role of insulin resistance in hyperuricemia and gout [127]. Metabolic factors related with T2DM, such as hyperinsulinemia, obesity, and hypertriglyceridemia, are strongly linked to hyperuricemia and gout [128,129]. Indeed, hyperinsulinemia was reported to reduce renal fractional excretion of urate via uncharacterized mechanisms [130]. A recent study found genetic interaction between SLC2A9 and its variants with human insulin, insulin receptor, and insulin receptor substrate-1 loci, which was most evident in females [131]. Notably, SLC2A9 genetic variants are more prominently associated with female gout, and SLC2A9-encoded GLUT9 may be involved in hyperinsulinemia associated with obesity and metabolic syndrome, which are more prevalent in female gout and promote hyperuricemia and gout.

CKD is clearly associated with hyperuricemia and sharply elevates the risk of incident gout [132]. CKD is more prevalent in females overall [133] and in female gout patients compared to male patients. When the 3-year cumulative incidence of gout was addressed, stratified by the level of eGFR, males showed a higher incidence of gout across all the levels of eGFR than females [134]. However, this likely reflects the higher prevalence of gout in males, rather than CKD contributing more to gout development in males. Indeed, with adjustment for confounding factors, CKD was associated with gout with an HR of 1.88 (1.13 to 3.13) among males and 2.31 (1.25 to 4.24) in females [135], a result which should not be interpreted as a greater prevalence of CKD in patients with gout. In females, owing to the overlapping 95% CIs.

Osteoarthritis (OA) is associated with gout, and the pathogenic link appears to extend beyond shared risk factors (e.g., age, obesity) to involve the effects of degenerative changes in cartilage and altered boundary lubricants in joints with OA [136,137]. In this context, we recently reported incident, erosive gouty arthritis without hyperuricemia in a young adult female with attenuated serum lubricin levels [10]. A decrease in lubricin promotes synovitis, and this study implicated TLR2 ligands in suppressing fibroblast-like synoviocyte lubricin levels. Moreover, lubricin, at concentrations present in normal joint fluids, was found to markedly suppress MSU crystal precipitation [10]. Lubricin also blunted the capacity of IL-1 to induce xanthine oxidase and elevated urate in synovial resident macrophages [10]. Lubricin is reduced in OA joints, which suggests a link between the increased risk of postmenopausal females for developing gout and OA. In addition, type II collagen, which is released from OA articular cartilage, can increase MSU crystallization in vitro [138] and enhance inflammatory responses to MSU crystals [9]. Prevalence of OA is higher in aged females. In nodal hand OA, which is more common in females, gout is commonly superimposed on distal interphalangeal joint OA [139]. Notably, OA patients with gout are at higher risk of total knee replacement surgery [140]. However, an MR study found no causal association between OA and gout [141]. Therefore, the link between OA and gout is likely not mediated by genetic association. Instead, changes in the articular cartilage surface and other changes in joint biology in OA (e.g., low grade synovitis, decreased synovial lubricin production [10,137], decreased hyaluronan production that also can dampen gouty inflammation [142], as well as increased type II collagen release from damaged cartilage) likely predispose individuals to intra-articular MSU crystal deposition and could impact intra-articular xanthine oxidase and urate production in the joint [143].

### 3.4. Genetic Studies

Genetic factors clearly influence serum urate level and gout [7]. Genome-wide association studies (GWAS) have revealed genetic variants associated with hyperuricemia, with effect sizes differing according to sex [7,70,88,115,144]. Two recent studies addressed genetic risk for gout using polygenic risk score (PRS), with female gout patients included. A gout PRS, calculated in large European and Polynesian cohorts, was associated with earlier age at gout onset and tophaceous disease in males but not in females [110]. In another study, a PRS for gout was determined in 59,472 Taiwanese and Chinese female gout patients, stratified by age to take the influence of menopause into account. Six variants located in SLC2A9, C5orf22, CNTNAP2, and GLRX5 were significant predictors of female gout in subjects ≥ 50 yrs. For those under 50 yrs, only the variant rs147750368 (SPANXN1) on chromosome X was found [47]. Results suggested that even females bearing gout risk gene variant alleles do not commonly develop gout until they are older, and that the genetic variants underlie a lower portion of incident gout in females compared to males.

## 4. Differences in Age and Clinical Characteristics of Gout in Females

The different clinical characteristics of female compared to male gout include onset of gout almost a decade later in females than males [6,55]. The onset age difference is associated with menopause and loss of aforementioned sex hormone protective effects. Female gout patients overall have more comorbidities associated with aging, such as hypertension, T2DM, and CKD. The higher prevalence of hypertension is not only related to more diuretic use but also impaired renal dysfunction, which is more prevalent in female gout patients. Female gout patients also have higher BMI, though they consume less alcohol than males [46,49,55,56]. the sites of clinically manifest gouty arthritis differ in females. The typical presentation of arthritis in the first toe metatarsophalangeal joint (podagra) is less frequent in females, who also tend to have oligoarticular presentation affecting other sites, such as small hand joints and the ankle [55]. Furthermore, gout can be superimposed on existing OA, which often leads to delayed diagnosis. Also, the degree of severity of many comorbidities in females that overshadow gout can contribute to delayed diagnoses. Mean serum urate level at diagnosis is higher in female gout patients (8.91 ± 2.19 mg/dL in females vs. 8.24 ± 1.85 mg/dL in males) [49].

A study from the Netherlands reported characteristics of 161 female and 793 male gout patients [49], specifically comparing patients ≥ 55 yrs to explore effects of sex hormones. Most of the differences were attenuated in the ≥55 yrs group, and after menopause, the gout phenotype was more similar to that of males.

Strikingly, females but not males with gout had an increased risk of COVID-19 infection and higher COVID-19-related death [145]. The higher risk of death for females with gout remained significant after adjusting for 16 other diseases, for BMI, and for age, though it is possible that this adjustment did not fully account for underlying metabolic disease diathesis in females with gout. Nevertheless, it remains possible that female gout itself, rather than gout-associated comorbidities, could be an independent risk factor for COVID-19, potentially via differences in immunity and inflammation in females.

## 5. Potential Sex Differences in Gouty Inflammation

Gouty inflammation is primarily driven by innate immunity [11], which serves as the first line of defense against pathogen-associated molecular patterns (PAMPs) and danger-associated molecular patterns (DAMPs). Potential factors affecting inflammatory response that may contribute to clinical differences between female and male gout are summarized in Table 3. Importantly, sex hormones can affect the immune system by changing the tissue milieu that immune cells encounter [146]. Innate immunity also can be influenced by intrinsic (host) and extrinsic (environmental) factors such as age and certain comorbidities [147]. As cited above, females are substantially older than males when diagnosed with gout and have distinctions in sites of arthritis, favoring degenerative hand arthritis, and less frequent polyarticular gouty arthritis flares [55].

Sex hormones including estrogen, progesterone, and testosterone directly impact the inflammatory capacity and functions of immune cells [146]. Comparison of female and male transcriptomes in whole blood has revealed a sex-specific immune transcriptome, and genes influenced by sex have been associated with responses to cytokines, type I interferon signaling, and rheumatoid arthritis [148].

Postmenopausal females have less pronounced sex-specific differences in gene expression, suggesting a role for estrogen in maintaining sex dimorphism in the blood transcriptome [148]. Estrogen receptors, ERα and ERβ, function as transcription factors by binding to estrogen response elements in gene promoters and regulating transcription in the presence of estrogen [146]. Low levels of estradiol increase the pro-inflammatory capacity of macrophages and monocytes in both humans and mice. Sex-specific open chromatin regions have been identified in murine macrophages, indicating a sex dimorphic immune epigenome, and menopause is linked with epigenetic changes [146].

The innate immune response can be influenced by mitochondria. Moreover, mitochondrial dysfunction, a central driver of aging, has been implicated in not only the pathogenesis and pathophysiology of gout, but also cardiovascular, metabolic, and renal comorbidities such as HTN, obesity, type 2 diabetes, and CKD [146,149–151]. Firstly, mitochondria are involved in signal transduction of downstream of pattern recognition receptors (PRRs). Intracellular signaling pathways of several PRRs physically interact with mitochondria and act as modulators of their function [149]. For instance, Toll-like receptors trigger the recruitment of mitochondria to macrophage phagosomes, where they release reactive oxygen species (ROS). Secondly, when mitochondrial damage occurs (such as an increase in mitochondrial membrane permeability), mitochondrial DNA (mtDNA) can be released into the cytosol or extracellular space. This mtDNA can be recognized as a DAMP by PRRs, leading to the initiation of a proinflammatory response. Third, mitochondrial signals, including from oxidative stress, are linked to NLRP3 inflammasome activation [152]. Fourth, mitochondrial sex dimorphisms are evident [149,150], supported by the distinct male and female sex hormones in regulating mitochondrial energy, oxidative phosphorylation, and Ca2+ homeostasis [149,150]. The effects of estrogen on mitochondrial function can vary depending on the tissue and context. In most tissues, particularly heart, kidney, and skeletal muscle, female mitochondria have been reported to have upregulated antioxidant capacity, respiratory function, and mitochondrial biogenesis capacity, with lower ROS production than male mitochondria [149,150].

In humans, estrogen treatment reverses the mitochondrial dysfunction associated with menopause by increasing the expression of PPAR-γ coactivator-1α (PGC1α), a master regulator of mitochondrial biogenesis and a coactivator of nuclear respiratory factor (NRF)1/NRF2. This leads to the upregulation of expression of mtDNA-specific transcription factors, including mitochondrial transcription factor A (TFAM) and mitochondrial transcription factor B1 and B2 (TFB1M and TFB2M), as well as expression of the antioxidant enzyme glutathione peroxidase and manganese sodium dismutase (SOD2) [149]. Estrogen plays a significant role in heart and kidney protection in premenopausal females by modulating renal mitochondrial bioenergetics during acute kidney injury, hypertension, and T2DM [149]. Decreased activation in certain female tissues of AMP-activated protein kinase (AMPK1) and SIRT1, which are crucial regulators of mitochondrial biogenesis and gouty inflammation [153], may be significant.

## 6. Treatment Responses in Females with Gout

Most gout clinical trials have been conducted with a vast majority (~9:1 or more) of male patients, with only a few studies that address the efficacy of gout medications in females [46]. However, post-hoc analyses suggest that ULT treatment response does not differ between the sexes [54]. That said, gout is associated with a moderately higher risk of fracture [40,154], and postmenopausal females have elevated risk of osteoporotic fracture. A recent study indicated that ULT that achieves the serum urate target reduces the risk of fracture in gout patients [155]. Therefore, postmenopausal gout patients with osteoporosis might benefit from treat-to-target ULT. In a recent questionnaire-based study that addressed illness perception of gout according to sex, females felt more disabled, and their pain scores were higher in acute gout flares [156]. Furthermore, allopurinol hypersensitivity syndrome incidence is higher in female gout patients, which may be partially related to a larger CKD population [157,158]. Also, it should be noted there are limitations in choosing anti-inflammatory drugs (e.g., non-steroidal anti-inflammatory drugs) for acute flares in the CKD population [46]. Collectively, pharmacologic treatment should take into account the special considerations for female gout along with the different non-pharmacologic approaches (e.g., impact of lifestyle modifications).

## 7. Special Consideration in Treatment of Female Gout: Pregnancy, Breastfeeding

Normal pregnancy increases serum urate levels. Although a relatively small number of female gout patients are at their reproductive ages, pregnancy or breastfeeding limits the treatment options for these patients [159,160]. For acute flares, clinicians generally use corticosteroids, colchicine, and/or NSAIDs. However, in pregnancy, NSAID use is not recommended in the third trimester, as it can result in premature closure of the ductus arteriosus of the fetus. Certain corticosteroids (e.g., dexamethasone) can cross the placenta and also are not recommended. Colchicine is generally not recommended due to unknown effects on the fetus; that said, safe use of colchicine during pregnancy in patients with familial Mediterranean fever has been reported [161]. Regarding the use of ULT, xanthine oxidase inhibitors including allopurinol and febuxostat cannot be employed during pregnancy due to potential teratogenicity [160]. Pegloticase safety data do not exist for pregnancy in humans. Taken together, there are no unequivocally safe drugs to decrease the serum urate level during pregnancy.

For breastfeeding during gout acute flares, NSAIDs and corticosteroids can be used. With regard to ULT, allopurinol can be used while breastfeeding, but it is secreted into breastmilk, and the infant should be closely monitored for possible adverse reactions such as hypersensitivity or cytopenia [162]. There are no reported data on using febuxostat in breastfeeding females.

## 8. Conclusions

Gout is common in females, though it remains concentrated among postmenopausal females. A primary driver for the rise of gout prevalence in females after menopause is the loss of female sex hormone effects on serum urate levels. However, it remains unclear what effects other than decreased estrogen-mediated uricosuria are contributory, with one possibility being altered gut urate transport. Understanding the mechanisms of observed sex differences in the progression and susceptibility to gout in males and females may help tailor more effective treatment. Multiple factors, exemplified by genetics, sex hormones, comorbidities, lifestyle, and distinct inflammation responses appear to contribute to differences in gout according to sex. For example, polymorphism in the gut and renal urate excretory transporter ABCG2 is more strongly associated with serum urate in males than females, whereas SLC2A9 polymorphisms are more strongly associated with serum urate in females than males. Obesity is more frequently linked with gout in females and compounds genetic susceptibility factors. Alcohol consumption is less related to risk of gout in females.

Though males and postmenopausal females tend to have similar profiles of primary gout, comorbidity studies have mostly been small, and conclusions for sex differences in gout have been inconsistent. OA is a risk factor for gout, and articular cartilage surface and other changes in articular biology in OA (e.g., low grade synovitis, decreased lubricin, type II collagen release from damaged cartilage) could predispose these individuals to MSU crystal deposition. In this light, OA is more common in older females than males. Certain comorbidities that cause hyperuricemia (e.g., hypertension, CKD) are also more prevalent in female gout, but only so in younger patients. Hence, younger patients who have lower serum urate levels due to the protective effects of estrogen commonly develop gout when they develop urate-elevating comorbidities, and many such comorbidities are more common in female gout patients. Though post hoc analyses show no difference in the treatment response between females and males with gouty arthritis, females appear to report more pain and disability during gout flares. Lastly, the higher frequency of CKD and of severe cutaneous hypersensitivity reaction to allopurinol could narrow urate-lowering therapy options in females.

## Figures and Tables

**Table 1.** Effects of sex hormones on urate transporters.

Urate Transporter or Transport Modulator (Function) [References]	Tissue Expression	Estrogen Effects	Progesterone Effects	Testosterone Effects
SLC22A12/URAT1 (reabsorption) [66,89]	RA	↓		↑
SLC2A9/GLUT9 (reabsorption) [62,66,67]	RB, RA	↓		↓
	RB, RA	↓		
ABCG2 (secretion) [66,68,82]	I	↑	(−)	
	H	↓		
SLC22A6/OAT1 (excretion) [90]	RB	↑		↓
SLC22A7/OAT2 (secretion) [91]	RB	↑		↓
ABCC2/MRP2 [83]	I, H	↑ (males) (−) (female)		↓ (male) (−) (female)
SMCT1, SMCT2 (modulators of URAT1 function) [66,89]	RA	(−)	↓	↑
SGLT2 (modulator of SLC2A9 and URAT1 function) [92]	RA	↑		↑

Abbreviations: sodium monocarboxylate cotransporter (SMCT), renal basolateral (RB), renal apical (RA) intestinal (I), hepatic (H), organic anion transporter (OAT).

**Table 2.** Sex differences of gout comorbidities as risk factors for gout development.

Comorbidities	Prevalence in Gout Patients	Causal Effect on Gout (Observed in MR or Cohort Studies)
HTN [12,13]	Higher in females	Inconsistent
T2DM [13,14].	Higher in females	Causal effect identified for insulin resistance but not T2DM per se
Obesity [15,16]	Higher in females	Positive causal effect Higher in females
CKD [17–20]	Higher in females	Positive causal effect Higher in females
HF [21–23]	Higher in females	Causal effects not identified to date
OA [24]	Not specifically reported	Causal effects suggested to be due to changes in lubricin, hyaluronan, and the cartilage extracellular matrix in OA

Abbreviations: Mendelian randomization (MR), hypertension (HTN), type 2 diabetes mellitus (T2DM), chronic kidney disease (CKD), heart failure (HF), osteoarthritis (OA).

**Table 3.** Potential factors affecting inflammatory response that could contribute to sex differences in gout.

Potential Factor	Effect on Immune Response
Transcription [25,26]	• Responses to cytokine, type I interferon signaling in immune cells. • Gene expression regulated by transcription factors ERα and ERβ
Mitochondria [27,28]	• Female mitochondria have higher antioxidant capacity with lower ROS production, which is related to less NLRP3 activation in gout. • Estrogen increases PGC1α and NRF1/2, leading to upregulation of TFAM, TFB1M/TFB2M, and SOD2, thereby alleviating mitochondrial dysfunction. • Decreased activation of AMPK signaling due to decreased estrogen aggravates mitochondrial dysfunction that is related to gouty inflammation.

Abbreviations: estrogen receptor (ER), reactive oxygen species (ROS), PPAR-γ coactivator-1α (PGC1α), nuclear respiratory factor (NRF), mitochondrial transcription factor A (TFAM), mitochondrial transcription factor B1 and B2 (TFB1M/TFB2M), manganese sodium dismutase (SOD2), AMP-activated protein kinase (AMPK).

## Data Availability

Not applicable.
